# Supplementary material for: Content-rich biological network constructed by mining PubMed abstracts
Source: BMC Bioinformatics. 2004 Oct 8;5:147. doi: 10.1186/1471-2105-5-147 (PMC528731; doi:10.1186/1471-2105-5-147)
Supplement: Additional File 2 — The original results of the above study (non-essential files are deleted to keep the file size under the limit set by BMC bioinformatics). [file 1471-2105-5-147-S2.bz2 › chilibotAdditionalFile2/dip20/472ID9195914E10538/html/IL8RA_CXCL1.html]

 


 **IL8RA** and **CXCL1** 
  
Found 47 abstracts in PubMed, retrieved 20.  
 

 What does Google say? 
 PDF only 
| .edu only 

---

**Interactive relationship** (e.g. stimulation, inhibition, etc)

**Neutral relationship**- CXCR1  [ **IL8RA** ]  and CXCR2 are receptors for ELR positive CXC chemokines which include growth regulated oncogene alpha GRO  [ **CXCL1** ]  alpha and interleukin 8 IL 8.  Ref: 11356265 Brain Res Dev Brain Res, 2001
- Together, these results show that a CXC chemokine such as GRO  [ **CXCL1** ]  alpha can promote malignant growth of murine squamous cell carcinoma by a host CXCR  [ **IL8RA** ]  2 dependent pathway.  Ref: 10918606 Oncogene, 2000

**Non-interactive relationship** (e.g. studied together, co-existance, homology, etc.)

- Melanoma growth stimulatory activity  [ **CXCL1** ]  growth related protein alpha MGSA  [ **CXCL1** ]  GROalpha, which binds CXCR2 but not CXCR1  [ **IL8RA** ] , was unable to either stimulate IL 8 secretion in MNCs or desensitize these cells to respond to immobilized IL 8.  Ref: 11076803 Am J Physiol Lung Cell Mol Physiol, 2000
- In addition, we demonstrate that compounds which inhibit the actions of IL 8 and GRO  [ **CXCL1** ]  alpha via their receptors, CXCR1  [ **IL8RA** ]  and CXCR2, also inhibit HIV 1 replication in both T lymphocytes and macrophages, indicating potential therapeutic uses for these compounds in HIV 1 infection and AIDS.  Ref: 11483765 J Virol, 2001
- Selective CXCR2 antagonists may be useful to block the Gro  [ **CXCL1** ]  alpha induced priming response whilst preserving neutrophil functions mediated by CXCR1  [ **IL8RA** ] , the alternative neutrophil receptor for interleukin 8.  Ref: 11589350 Eur Respir J, 2001
- PMNs were exposed to interleukin 8 IL 8, growth regulated oncogene alpha GRO  [ **CXCL1** ]  alpha, and platelet activating factor PAF to stimulate the CXCR1  [ **IL8RA** ] , CXCR2, and PAF receptors.  Ref: 11130495 J Trauma, 2000
- For example strong calcium responses were seen in neutrophils following stimulation with the CXCR1  [ **IL8RA** ]  and CXCR2 ligands, interleukin IL 8, GCP 2 and Gro  [ **CXCL1** ]  beta.  Ref: 11298490 Cytokine, 2001
